# Integrating Network Pharmacology and Molecular Docking Approaches to Decipher the Multi-Target Pharmacological Mechanism of *Abrus precatorius* L. Acting on Diabetes

**DOI:** 10.3390/ph15040414

**Published:** 2022-03-29

**Authors:** Fatima Noor, Abdur Rehman, Usman Ali Ashfaq, Muhammad Hamzah Saleem, Mohammad K. Okla, Abdulrahman Al-Hashimi, Hamada AbdElgawad, Sidra Aslam

**Affiliations:** 1 Department of Bioinformatics and Biotechnology, Government College University Faisalabad, Faisalabad 38000, Pakistan; fatimanoor23@gcuf.edu.pk (F.N.); abdurrehman93@gcuf.edu.pk (A.R.); ashfaqua@gcuf.edu.pk (U.A.A.); 2 College of Plant Science and Technology, Huazhong Agricultural University, Wuhan 430070, China; saleemhamza312@webmail.hzau.edu.cn; 3 Department of Botany and Microbiology, College of Science, King Saud University, Riyadh 11451, Saudi Arabia; malokla@ksu.edu.sa (M.K.O.); aalhashimi@ksu.edu.sa (A.A.-H.); 4 Integrated Molecular Plant Physiology Research, Department of Biology, University of Antwerp, 2000 Antwerpen, Belgium; hamada.abdelgawad@uantwerpen.be

**Keywords:** active ingredients, *Abrus precatorius*, bioinformatics, network pharmacology, molecular docking

## Abstract

Type 2 diabetes mellitus (T2DM) is a notable health care load that imposes a serious impact on the quality of life of patients. The small amount of reported data and multiple spectra of pathophysiological mechanisms of T2DM make it a challenging task and serious economic burden in health care management. *Abrus precatorius* L. is a slender, perennial, deciduous, and woody twining plant used in various regions of Asia to treat a variety of ailments, including diabetes mellitus. Various in vitro studies revealed the therapeutic significance of *A. precatorius* against diabetes. However, the exact molecular mechanism remains unclarified. In the present study, a network pharmacology technique was employed to uncover the active ingredients, their potential targets, and signaling pathways in *A. precatorius* for the treatment of T2DM. In the framework of this study, we explored the active ingredient–target–pathway network and figured out that abrectorin, abrusin, abrisapogenol J, sophoradiol, cholanoic acid, precatorine, and cycloartenol decisively contributed to the development of T2DM by affecting AKT1, MAPK3, TNFalpha, and MAPK1 genes. Later, molecular docking was employed to validate the successful activity of the active compounds against potential targets. Lastly, we conclude that four highly active constituents, namely, abrusin, abrisapogenol J, precatorine, and cycloartenol, help in improving the body’s sensitivity to insulin and regulate the expression of AKT1, MAPK3, TNFalpha, and MAPK1, which may act as potential therapeutic targets of T2DM. Integrated network pharmacology and docking analysis revealed that *A. precatorius* exerted a promising preventive effect on T2DM by acting on diabetes-associated signaling pathways. This provides a basis to understand the mechanism of the anti-diabetes activity of *A. precatorius*.

## 1. Introduction

Type 2 diabetes mellitus (T2DM) is the most common form of diabetes mellitus and is a rapidly growing global problem [1]. T2DM is associated with the diverse interplay of genetic, environmental, and behavioral risk factors [2]. T2DM patients are often more vulnerable to a number of short- and long-term difficulties, which can result in serious complications [3]. In 2011, 366 million individuals were expected to have diabetes; by 2030, that number will have increased to 552 million [4]. It is proposed that the incidence of T2DM will rise in the next couple of decades, with much of the growth appearing in the developed world [5]. Despite the fact that the etiology and pathogenesis of T2DM are unclear, insulin resistance has long been thought to be a major pathological hallmark of T2DM patients [6,7]. As a result, understanding the mechanisms of insulin resistance and discovering novel drugs that promote insulin sensitivity are critical in the fight against T2DM. Pharmacotherapy consisting of natural products is seen as a viable therapeutic method for T2DM and could provide answers to the questions raised above.

*Abrus precatorius* L. is a herbaceous plant of fabaceae, which flowers in winters. The latest pharmacological studies suggest that *A. precatorius* possesses antidiabetic, antimicrobial, anticancer, and anti-inflammatory properties due to the presence of natural bioactive compounds [8,9]. Alkaloids, steroids, tannin, triterpenoids, protein, flavonoids, and phenolic compounds are among the secondary chemicals isolated from *A. precatorius* [8,9,10,11]. The findings of this study show that *A. precatorius* is a root and distinctive source of numerous promising phytochemicals, making it a valuable and adaptable plant with a wide range of therapeutic qualities.

Network pharmacology (NP) is a rising star in the field of drug discovery, as it integrates systematic medicine with information science [12]. It is an integrative in silico approach for introducing a ‘protein-compound/disease-gene’ network in order to reveal the mechanisms underlying the synergistic therapeutic actions of traditional medicines [13]. This advancement, in turn, has shifted the paradigm from a ‘one-target, one-drug’ mode to a ‘network-target, multiple-component-therapeutics’ mode.

The use of bioactive compounds to reform medicines in the future is exciting, and prospects for curing multiple diseases are encouraging. Recently, Li et al. [14] employed a network pharmacology-based approach to explore the active ingredients of Ge-Gen-Qin-Lian decoction for the treatment of T2DM. Hence, network pharmacology provided a powerful means for identifying bioactive ingredients and mechanisms of action for Traditional Chinese medicinal (TCM) herbs/herbal formulae for the treatment of disease and disorders [15]. In this current work, a comprehensive NP-based approach was used to explore the active ingredients of *A. precatorius*. To the best of our knowledge, this is the first study that integrated bioinformatics analysis with NP to explore the mechanism of *A. precatorius* for T2DM treatment. This study gives a new and novel insight in terms of understanding the molecular mechanism of the anti-diabetic activity of *A. precatorius* and expedites the process of drug discovery. Moreover, this breakthrough has sparked a new interest in the search for candidate drugs from *A. precatorius*. In the framework of this study, bioactive compounds of *A. precatorius*, as well as the putative mechanism underlying its anti-T2DM effect, were analyzed by employing a network pharmacology approach along with molecular-docking analysis. Moreover, laboratory experiments are recommended to explore the substance’s pharmacological potential in the near future.

## 2. Results

### 2.1. Screening of Active Compounds and Targets

After searching, filtering, and removal of the duplicates, 11 putative components (Abrisapogenol J, Precatorine, Sophoradiol, Abrectorin, Isoorientin, Cholanoic acid, Cycloartenol, Amyrin, luteolin, Skrofulein, and Abrusin) with F ≥ 30% and DL ≥ 0.18 were selected (Table 1). F30 means that the bioavailability is 30%. Bioavailability is the rate and extent to which the active constituent or active moiety of a drug is absorbed from a drug product and reaches the circulation [16]. Compounds that are formulated to have high bioavailability will be more effective, as they will help the body to absorb more of the appropriate nutrient without having to take higher doses. Drug likeness (DL) measures the likelihood of a chemical becoming an oral drug in terms of bioavailability. DL derived from structures and properties of existing drugs and drug candidates has been widely used to filter out undesirable compounds in the early phases of drug discovery [17]. Further, 456 potential target genes of 11 active constituents were retrieved from the Swiss Target Prediction database. After identifying the promising targets of compounds, a total of 11,563 genes affiliated with T2DM were retrieved from GeneCards and OMIM databases. Later, the common targets of both T2DM and the compound-related genes were predicted through a Venn diagram. A total of 397 potential anti-T2DM genes of *A. precatorius* were selected and considered as key targets.

### 2.2. Compounds—Target Network Construction

A total of 11 satisfactory active compounds were obtained from *A. precatorius*. Further, 11 active compounds, 397 key targets, and their associated pathways with a maximum number of genes were chosen for the construction of an ‘active compound–targeted genes–connected pathway’ network diagram. Each of these active compounds corresponded to multiple targets. This is a strong indication that many targets may induce a synergistic effect when *A. precatorius* serves as an anti-type 2 diabetic agent. The degree of these 11 compounds in the compound–targeted genes–connected pathways network was then evaluated (Table 2). As highlighted in Table 2, triterpenoids, along with flavonoids, had the highest degree of connectivity, but the degree of both alkaloids and steroids was comparatively low compared to triterpenoids and flavonoids. Further, from these 11 compounds, 7 compounds were selected for docking analysis: 2 flavonoids with the highest degree of connectivity, particularly abrectorin and abrusin; 3 triterpenoid components, namely, abrisapogenol J, sophoradiol, and cholanoic acid; 1 alkaloid, namely, precatorine; and 1 steroid component, namely, cycloartenol.

### 2.3. PPI Network Construction

**Figure 1:**
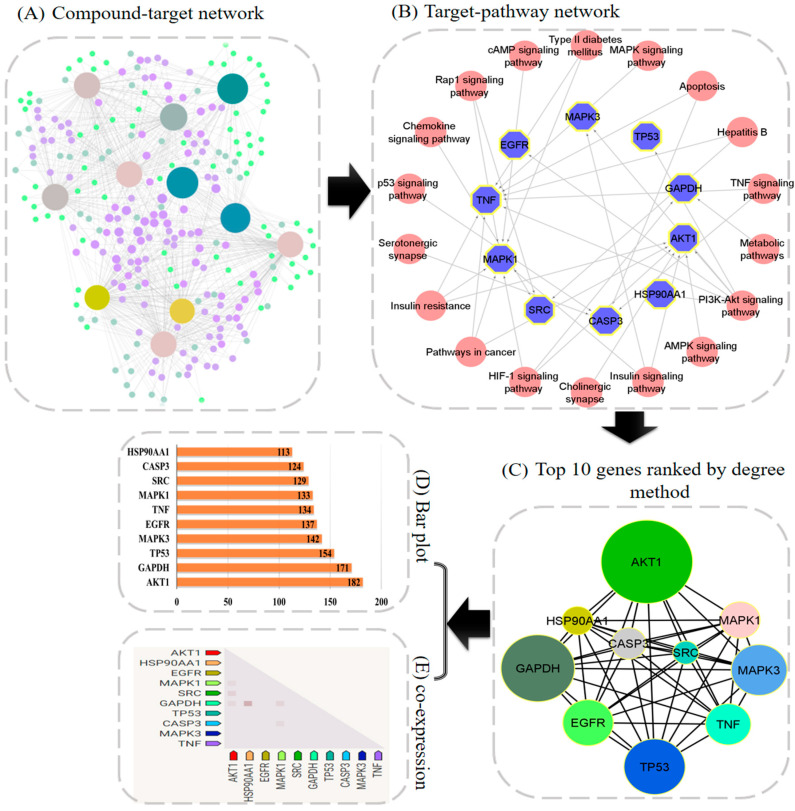
Network-pharmacology-based analysis of multi-compound, multi-target, and multi-pathway treatment for T2DM. (**A**) Network diagram of compounds and their targets. (**B**) Network diagram of target genes–enrichment pathways. The blue octagon indicates the targets, and pink nodes indicate the pathways. (**C**) Top 10 genes ranked by degree. (**D**) The bar plot of the PPI network. (**E**) Observed expression of 10 target genes in *Homo sapiens*.

The 397 overlapped genes were submitted into the STRING database for the construction of the PPI network. In the PPI network, nodes and their associated interactions indicate the interrelationship among multiple targets during disease development (Figure 1A). Later, a network analyzer tool was employed for analyzing the PPI network of overlapped genes (Figure 1C). AKT1 (182), GAPDH (171), TP53 (154), MAPK3 (142), EGFR (137), TNFalpha (134), MAPK1 (133), SRC (129), CASP3 (124), and HSP90AA1 (113) showed the highest degrees of overlapping (Figure 1D). The highest degree means that targeted genes are greatly correlated with each other; hence, all these genes might be key targets. After comparing these findings with those supplied by enrichment analysis (Table 3), four genes, particularly AKT1, MAPK3, TNFalpha, and MAPK1, were identified as the main anti-T2DM targets of *A. precatorius* and were chosen for molecular-docking experiments.

### 2.4. GO and KEGG Analysis

**Figure 2:**
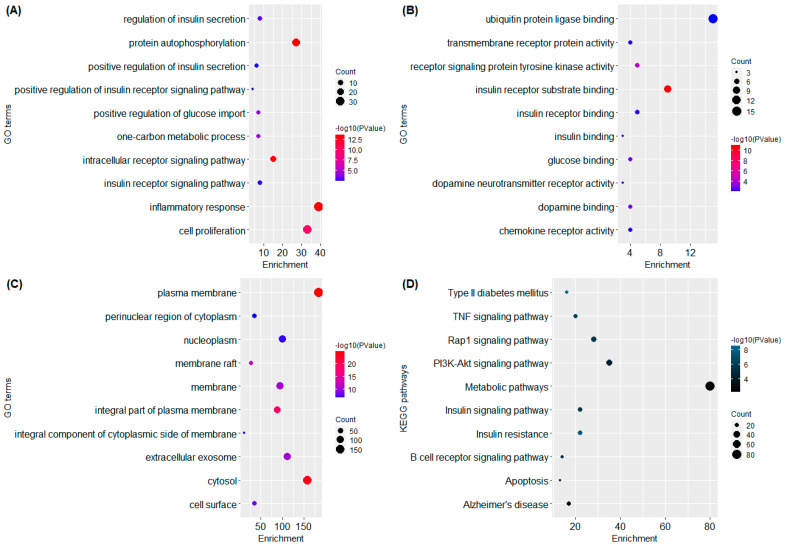
Representation of functional annotation and enriched pathways in form of Bubble Plot. (**A**) GO in terms of biological processes. (**B**) GO in terms of molecular function. (**C**) GO in terms of cellular components. (**D**) KEGG pathway analysis.

**Figure 3:**
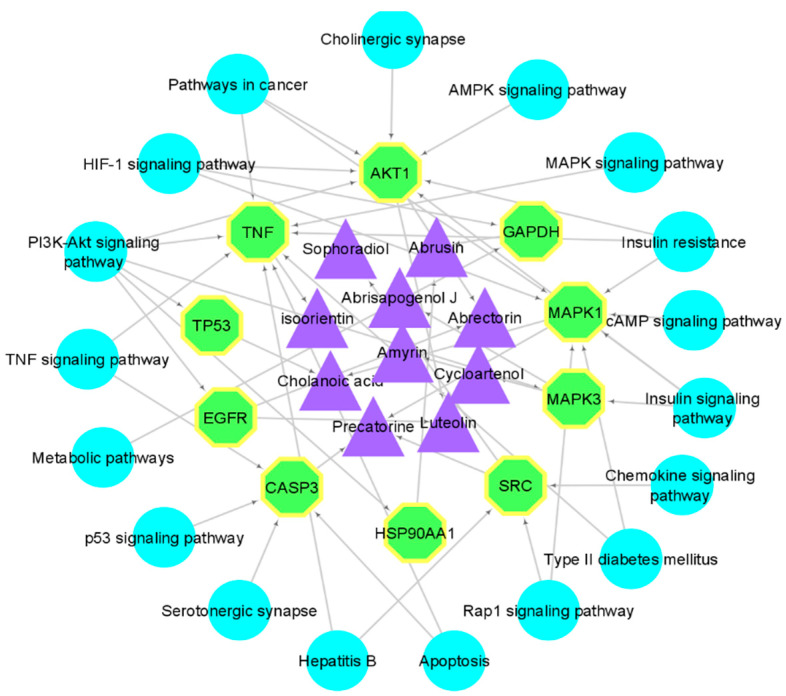
Pathways influenced by *A. precatorius*. The green nodes represent the hub genes, the purple nodes represent active compounds, and the blue nodes are the pathways associated with the core targets.

The functional annotation and enrichment analysis revealed potential biological functions of *A. precatorius* targets. According to GO functional analysis, *A. precatorius* targets were related to protein autophosphorylation, insulin receptor substrate binding, the regulation of insulin secretion, and so forth (Figure 2). The KEGG pathway analysis was performed to identify the significant signaling pathways linked to the anti-T2DM effect of *A. precatorius*. It is noteworthy that most of the genes were involved in the following pathways: neuroactive ligand–receptor interactions (47), insulin resistance (22), type II diabetes mellitus (16), and pathways in cancer (55). Finally, KEGG pathway analysis revealed that AKT1, MAPK3, TNFalpha, and MAPK1 were significantly enriched genes (Figure 3).

### 2.5. Molecular Docking

**Figure 4:**
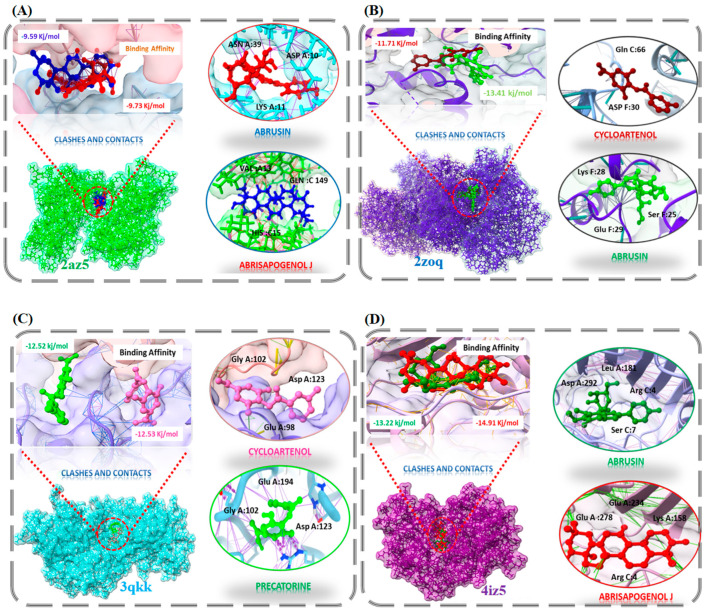
The docked complexes of four gene along with their strongest binding compounds. (**A**) TNFalpha, (**B**) MAPK3, (**C**) AKT1, (**D**) MAPK1.

Through systematic analysis of the PPI network, the top four compounds, named MAPK1, MAPK3, TNFalpha, and AKT1, were selected for molecular docking. The crystal structure of target proteins (MAPK1 (PDB id: 4IZ5), MAPK3 (PDB id: 2ZOQ), TNFalpha (PDB id: 2AZ5), and AKT1 (PDB id: 3QKK)) were retrieved from PDB. Structural refinement was completed by using the ucsf chimera tool. Energy minimization was completed at 1000 decent steps, while the non-standard residues were also removed from the receptors of the protein to avoid clashes and incorrect configurations. Molecular docking was performed to screen out the putative targets of constituents with the ability to lower the risk of T2DM. Docking analysis successfully predicted the strong binding affinity between constituents and the binding pockets of four target proteins. Docking scores, along with binding energy, were employed as key criteria for compound screening (Table 4). Clusters having a maximum absolute value of binding energy and highest conformation were selected. 2az5 had the maximum binding energy and RMSD with abrisapogenol J and abrusin, 2zoq has the highest RMSD and binding energy with cycloartenol and precatorine, 3qkk has the maximum binding energy and RMSD with abrisapogenol J and abrusin, and 4iz5 binds stably with abrusin and cycloartenol. Hence, these results imply that active constituents of *A. precatorius* bind stably with four target proteins and function as a T2DM repressor. Moreover, Thalidomide [18], Minocycline [18], Resveratrol [19], and Ulixertinib [20] were identified as positive control drugs of TNFalpha, MAPK3, AKT1, and MAPK1, respectively. Molecular-docking analysis demonstrated that abrisapogenol J, abrusin, cycloartenol, and precatorine showed stronger binding energies with the target protein compared to positive control drugs (Table 5). This fact revealed the validity of the present work. Additionally, understanding the interaction between these four targets is core to deeply understanding the mechanism of action of active constituents against T2DM. All the drug candidates showed hydrogen bond, Pi–pi-stacked, and van der Waals interactions with the receptor proteins, indicated with dotted lines mentioned in the additional file (Supplementary File, Table S1). Furthermore, hydrogen bonds interactions with receptor proteins are mentioned in Figure 4. Overall, these findings add to a new growing body of evidence suggesting that these four proteins are putative targets of *A. precatorius* for the treatment of T2DM. Moreover, the binding pockets of active constituents with the core protein will become the focus of further study. Figure 4 represents the sketch maps of target proteins together with their strongest binding components.

### 2.6. ADMET Profiling

ADMET analysis is a challenging process in drug discovery. This is achieved through SwissADME database and showed that selected compounds have good pharmacokinetic properties. ADMET profiling of all those top selected drug candidates shows that there is no side effect of pharmacokinetic properties of all potential compounds (Table 6). The associated ADMET properties of the potential compounds for different models, such as P-glycoprotein substrates, BBB penetration, and gastrointestinal absorption, showed positive results that strongly support the compounds’ ability to function as a drug candidate. All the compounds showed non-toxic behavior, although different types of toxicity were measured for all compounds, and none of the compounds showed toxic behavior.

## 3. Discussion

Type 2 diabetes mellitus is a multifactorial chronic metabolic condition characterized by relative insulin deficiency, hyperglycemia, and insulin resistance [21]. All around the globe, the rate of patients with diabetes mellitus has reached an unimaginable level, and 80 percent of those with the disease reside in low- and middle-income countries [22]. Metformin, Repaglinide, Sitagliptin, Glimepiride, Pioglitazone, Sitagliptin, and Acarbose [23] are generally prescribed for T2DM patients, but all these oral anti-diabetic medicines have been linked to major side effects. As a result, the search for new medications has grown more focused. In this context, a high-potency source of phytoconstituents with health advantages could be a promising T2DM treatment candidate.

Medicinal plants are considered a natural pool and an infinite source of medicinal agents due to the presence of naturally occurring compounds [24,25]. Natural products and their derivatives make up nearly half of all clinically used pharmaceuticals, and due to their structural variety, multi-target action, and low toxic side effects, they have been a hot topic in recent years as a research trend and possible source for targeted drugs. Over the past dozen years, high-throughput techniques have proposed a strong arm in screening the pharmacological efficacy of herbal medicines in drug discovery [26,27]. The discovery of potentially bioactive compounds that cease the pathophysiology of disorders and diseases will be considered a thunderbolt of this present epoch.

*A. precatorius* is a medicinal herb abundantly found across Afro-Asian regions of the world. This plant has therapeutic properties used to treat a variety of ailments. Various parts of the plant, such as seeds, roots, and leaves, are utilized for a variety of medical purposes. Flavonoids, glycosides, triterpenoids, abrin, and alkaloids are the main components of *A. precatorius* [8,28,29]. The conventional usage of *A. precatorius* in the treatment and management of diabetes mellitus has been emphasized in various reports [30,31]. Of note, compounds of *A. precatorius* revealed efficacy in treating breast cancer [32] and diabetes mellitus [33]. This study is a touchstone for the initial screening of bioactive compounds of *A. precatorius* as well as a new therapeutic concept for further exploration on mechanisms of *A. precatorius* for T2DM treatment. In our line of work, screening results represented that triterpenoids, flavonoids, steroids, and alkaloids were the main bioactive compound of *A. precatorius*, which played a decisive role in the development of T2DM by affecting AKT1, MAPK3, TNFalpha, and MAPK1 genes. Furthermore, molecular docking also confirmed our findings that stable binding forces exist between core compounds and key targets. By constructing a model of ‘herb-active compounds–targets–pathways’, we discovered that abrectorin, abrusin, abrisapogenol J, sophoradiol, cholanoic acid, precatorine, and cycloartenol had a strong association in the network, indicating that it has anti-diabetic properties. Moreover, molecular docking also strengthened our findings by successfully validating the interaction that exists between highly active constituents and their putative targets. Finally, the associated ADMET properties of potential compounds for different models, such as P-glycoprotein substrates, BBB penetration, and gastrointestinal absorption, showed positive results that strongly support the compounds’ suitability as drug candidates.

According to GO functional analysis, the anti-diabetic targets of *A. precatorius* were mainly involved in insulin receptor substrate binding, protein autophosphorylation, insulin receptor substrate binding, and the regulation of insulin secretion. KEGG pathway studies revealed that targets were concentrated in diabetes-related pathways. In addition to being enriched in diabetes-related pathways, anti-diabetic targets were also involved in other pathways that are intimately tied with T2DM, such as neuroactive ligand–receptor interaction, the AMPK signaling pathway, the PI3K-Akt signaling pathway, and pathways in cancer.

In the current analysis, we discovered many target genes participating in various metabolic pathways. By targeting the genes that cause disturbance in metabolic pathways, the pathophysiology of disease can be halted. A few of studies strengthened our findings, such as one that showed that most people suffering from T2DM are insulin resistant just because of glucose toxicity [34]. It is noteworthy that two of our key targets, namely, AKT1 and MAPK1, are mainly involved in insulin-resistant pathways. AKT1 or Akt serine/threonine kinase 1 regulates glucose metabolism [35]. This gives clear evidence to the concept that the dysregulation of AKT1 signaling pathways may be affiliated with an increased risk of T2DM. In the wake of the worldwide increase in T2DM, the central theme of research is to better understand the signaling pathways affecting this disease. Our analysis proposed that AKT1, MAPK1, and MAPK3 are directly involved in insulin signaling pathways. Hence, variation in these genes may cause disturbance in the associated pathways, which in turn leads to a disease state. Beyond this, the targeted genes of active constituents are also enriched in various inflammatory conditions such as rheumatoid arthritis, which seems to indicate that it can act on various anti-inflammatory cytokines and exert an effect on T2DM.

According to the ‘compounds–targets network’, we screened seven compounds and four genes for a docking experiment. Furthermore, docking results verified our findings and showed that abrisapogenol J, abrusin, cycloartenol, and precatorine bind stably with the active pockets of target genes, which spotlight that these compounds can be considered for the treatment of T2DM by inhibiting AKT1, MAPK3, TNFalpha, and MAPK1 genes. In the light of network pharmacology, the current study elaborates on the active compounds, their potential targets, and associated pathways to treat T2DM, hence providing a theoretical basis for additional experimental research. Bearing in mind the limitations of network pharmacology, the basic pharmacological mechanisms for the treatment of T2DM is only achieved by mining data. Network pharmacology presently relies on different databases for bioactive mining. Databases, though curated, may show discrepancies due to numerous sources of information and experimental data. A way to navigate around this problem is to make use of modern, high-throughput chemical identification techniques such as ultra-performance liquid chromatography–electrospray mass spectroscopy [36]. Despite the fact that we have presented some interesting data, additional studies and clinical trials are needed to explore the potential of *A. precatorius* to validate their medicinal usages.

## 4. Materials and Methods

### 4.1. Collection and Screening of Active Compounds

Information on active constituents of *A. precatorius* was retrieved from literature as well as database of biologically active phytochemicals (Indian Medicinal Plants, Phytochemistry Additionally, Therapeutics (IMPPAT) [37], traditional Chinese medicine systems pharmacology (TCMSP) [38], and KNApSAcK [39]) were used to collect the active constituents of *A. precatorius*. ‘*Abrus precatorius*’ was used as a keyword in databases, while a literature search was carried out on Pubmed and Google Scholar. All ingredients of *A. precatorius* were virtually screened by applying bioavailability (F) and drug-likeness (DL) parameters, which are crucial in the characteristics of absorption, distribution, metabolism, and excretion (ADME) characteristics of drugs. Ingredients were only retained if DL ≥ 0.18 and F ≥ 30% to satisfy ADME criteria. In this regard, F30% and DL of all active compounds were calculated using SwissADME and ADMETlab. Meanwhile, chemical information (CID number, structure, and molecular weight) of screened compounds was also collected from PubChem and ChemSpider.

### 4.2. Screening for Potential Target Genes for A. precatorius Active Constituents against T2DM

Putative targets of the selected compounds of *A. precatorius* were predicted using Swiss Target Prediction [40] and STITCH databases [41]. Screened compounds were uploaded to STITCH database in order to achieve the targets with the search limited to ‘Homo sapiens’. Only targets with a combined score of ≥0.7 were selected for subsequent analysis, while SMILES number of each constituent was used in the online platform of Swiss Target Prediction to obtain targets using reverse pharmacophore matching approach. Therefore, the targets with probability ≥0.7 were selected.

Prediction of disease-related genes is the next preliminary step to explore the molecular mechanism of medicinal herbs to treat multiple diseases and disorders. Two databases, GeneCard [42] and Online Mendelian Inheritance in Man (OMIM), were searched with keywords ‘Type 2 diabetes mellitus’ and ‘T2DM’ to retrieve disease-related genes. In addition, these databases also provided concise genomic information along with functional annotation of known human genes. All duplicated genes were discarded from the final gene list, and UniProtKB [43] was employed to obtain the standard name of the target gene, with the organism selected as ‘Homo sapiens’. Then, the predicted target genes of screened *A. precatorius* compounds and T2DM-related targets were intersected, and Venn plot was drawn to extract the common targets for subsequent analysis.

### 4.3. Pathway and Functional Enrichment Analysis

Database for annotation, visualization, and integrated discovery (DAVID) was hired to perform functional annotation and enrichment analysis [44]. The key targets were subjected to DAVID for the prediction of function at three levels: biological process (BP), molecular function (MF), and cellular component (CC). In this study, adjusted *p*-value  ≤  0.05 was selected, and top 10 GO enrichment and top 10 KEGG pathways with higher counts were chosen for further analysis.

### 4.4. Network Construction

Network analysis was performed to understand the mechanism of *A. precatorius* in T2DM. The network was constructed and visualized by Cytoscape 3.8.0, which is a freely available graphical user interface for importing, visually exploring, and analyzing biomolecular interaction networks. Nodes were used to represent the active constituents and target genes in the network, while interactions between active constituents and their target genes were indicated by edges. Network analyzer tool was used to calculate degree, a topological property that reveals the importance of compound/target gene/pathways in network diagram. Further, target genes with the highest degree of connectivity were considered as ‘key targets’.

### 4.5. Protein–Protein Network Construction and Molecular Docking

Protein–protein interactions (PPI) are highly significant by virtue of having high versatility, adaptability, and specificity [45,46,47]. Search Tool for the Retrieval of Interacting Genes/Proteins (STRING) was used to determine the functional interactions among key targets with a combined score of more than 0.4 [48]. The PPI network obtained from STRING was subjected to the CytoHubba plugin of cytoscape, which was used to analyze the core regulatory genes of the PPI network and the identification of key targets.

Furthermore, key targets were validated through molecular docking approach. The RCSB Protein Data Bank was used to obtain the X-ray crystal structures of candidate targets [49]. PDB is a single worldwide archive containing information related to the 3D structures of proteins and nucleic acids. In addition, refinement of structure was completed using Chimera [50]. Then, site finder tool from molecular operating environment (MOE) was used to find binding pockets of target proteins [51], and PyRx software was employed to perform target docking among core targets and active compounds [52]. The best-docked poses with the lowest root mean square deviation (RMSD) and binding energy were selected for further analysis. Docking scores between core target and compounds were utilized as key evaluation criteria to filter out potential constituents and their putative targets. For each docked complex, the model with maximum absolute value of binding energy and highest were considered accurate. Moreover, Chimera X [53] and discovery studio [54] were used for visualization of interaction among active compounds and predicted proteins/targets.

### 4.6. ADMET Profiling

**Figure 5:**
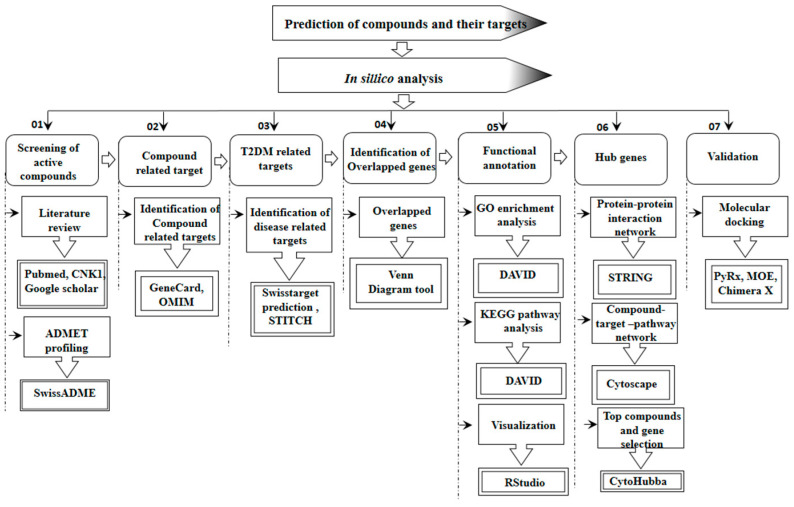
Graphical synopsis representing the overall strategy used in the prediction of potential compounds and their potential targets for T2DM treatment.

SWISS ADME online server was used to check the physiochemical properties of compounds, including absorption, distribution, metabolism, excretion, and toxicity [17]. All those properties influence the levels of drug or kinetics of drug revelation to the tissues and therefore influence the pharmacological activity and performance of the compound as a medication [55]. Good-quality drug molecules should have adequate efficacy against the therapeutic targets and indicate appropriate ADMET properties at a therapeutic dose [56]. ADMET predictor is a machine learning software that reliably predicts the best drug candidates by passing 175 parameters, including solubility, logp, and pKa sites of CYP metabolism [57]. Protox II tool was used for the toxicity prediction of different types of toxicity, such as cytoxicity, mutagenicity, AMES, and carcenogenes. The workflow of the present study is displayed in Figure 5.

## 5. Conclusions

This research establishes the latest scientific foundation for determining the efficacy of multi-component, multi-target compound formulas, together with exploring more therapeutic targets for T2DM. In this study, we incorporated a network pharmacology approach with molecular docking in order to uncover the molecular mechanisms of *A. precatorius* for the treatment of T2DM. Further, our findings propose that AKT1, MAPK3, TNFalpha, and MAPK1 genes are promising and viable therapeutic targets to reduce the incidence of T2DM, thereby exerting potential therapeutic effects in T2DM. However, this study also has certain limitations, as pharmacological and clinical research still need to further validate our findings. This approach introduces a groundwork for further research on the protective mechanisms of *A. precatorius* for T2DM and applications of network pharmacology in drug discovery.

## Figures and Tables

**Table 1:** Active compounds, their properties, and structures.

Molecule Name	Molecular Weight (MW)	Drug Likeness (DL)	Bioavailability (F30%)	Structure	PubChem ID
Abrisapogenol J	456.78	0.74	0.82	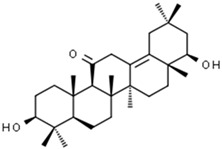	21594179
Precatorine	289.26	0.19	0.976	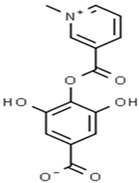	54704420
Sophoradiol	442.8	0.76	0.745	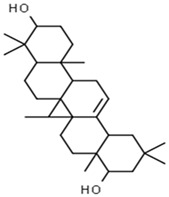	9846221
Abrectorin	314.31	0.31	0.978	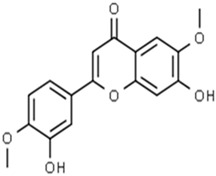	44257585
Isoorientin	448.41	0.76	1	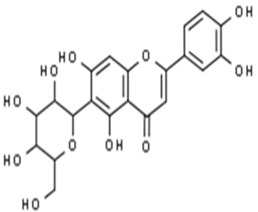	114776
Cholanoic acid	360.64	0.59	0.919	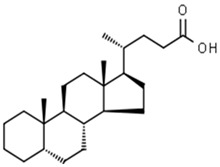	92803
Cycloartenol	426.8	0.78	0.972	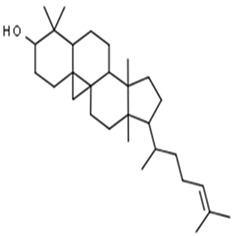	92110
Amyrin	426.8	0.76	0.877	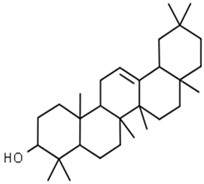	73145
luteolin	286.25	0.25	1	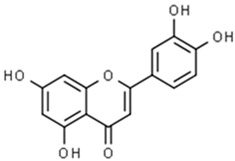	5280445
Skrofulein	314.31	0.3	0.632	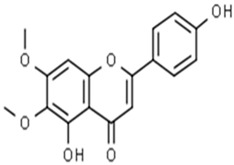	188323
Abrusin	476.47	0.78	0.905	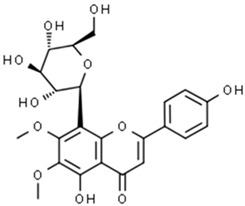	44258417

**Table 2:** Degree of 11 compounds explored through network analyzer in Cytoscape.

Molecule Name	Class	Degree
Abrectorin	Flavonoids	58
Abrusin	Flavonoids	45
isoorientin	Flavonoids	15
Skrofulein	Flavonoids	5
Luteolin	Flavonoids	4
Abrisapogenol J	Triterpenoids	94
Cholanoic acid	Triterpenoids	40
Sophoradiol	Triterpenoids	36
Amyrin	Triterpenoids	7
Precatorine	Alkaloids	78
Cycloartenol	Steroids	16

**Table 3:** Top 10 genes ranked by degree.

Gene Name	Compounds	Score	Pathways
AKT1	Abrectorin/isoorientin/Luteolin	182	AMPK signaling pathway, insulin resistance, PI3K-Akt signaling pathway, insulin signaling pathway
GAPDH	Abrusin	171	Metabolic pathways
TP53	Cholanoic acid	154	PI3K-Akt signaling pathway
MAPK3	Abrisapogenol J/Cycloartenol/Amyrin	142	PI3K-Akt signaling pathway, insulin signaling pathway
EGFR	Abrectorin/isoorientin/Luteolin	137	PI3K-Akt signaling pathway
TNFalpha	Isoorientin	134	Type II diabetes mellitus, insulin resistance
MAPK1	Precatorine/Cholanoic acid	133	Type II diabetes mellitus, insulin resistance, insulin signaling pathway
SRC	Precatorine/sophoradiol	129	Rap1 signaling pathway
CASP3	Precatorine	124	p53 signaling pathway
HSP90AA1	Abrusin	113	PI3K-Akt signaling pathway

**Table 4:** Binding energy and interactions of potential active compounds and their four target proteins.

Target Proteins (PDB ID)	Compounds	Binding Affinity (kcal/mol)	RMSD	Interacting Residues
2az5	Abrisapogenol J	−9.7335	1.45	HIS C: 15, LEU A: 36, VAL C: 17, ALA A: 38, LYS A: 11, ASP A: 10, ASN A: 39, ILE A: 155, TYR C: 151
	Abrusin	−9.5991	2.02	HIS C: 15, LEU A: 36, VAL A: 13, LEU C: 36, ASP A: 100, GLN C: 150
2zoq	Cycloartenol	−12.529	1.32	GLY A: 102, ASP A: 123, LYS A: 181, ARG A: 104, HIS B: 195
	Precatorine	−12.527	1.32	GLY A: 102, ASP A: 123, LYS A: 181, ARG A: 104, HIS B: 195
3qkk	Abrisapogenol J	−13.22	0.84	LEU A: 295, LEU A: 181,PHE A: 161, LYS A: 158, PHE A: 442, VAL A: 164, GLU A: 278, GLU A: 234, ARG C: 4
	Abrusin	−14.91	1.93	LEU A: 181,LYS A: 179, ASP A: 292, THR A: 291, SER C: 7, LYS A: 276, ARG C: 4
4iz5	Abrusin	−13.41	1.32	SER F: 70, SER F: 25, ALA F: 26, GLY C: 182, LYS F: 28, GLU F: 29, THR C: 181
	Cycloartenol	−11.716	1.92	GLN C: 66, ASP F: 30

**Table 5:** Binding energy and interactions of control drugs.

Target Protein	Control Drug	PubChem ID	Binding Energy	RMSD
TNFalpha	Thalidomide	5426	−6.9	1.3
MAPK3	Minocycline	54675783	−6.7	1.9
AKT1	Resveratrol	445154	−5.9	1.8
MAPK1	Ulixertinib	11719003	−6.2	3.48

**Table 6:** ADMET profiling of compounds.

Compounds	Abrisapogenol J	Abrusin	Precatorine	Cycloartenol
GI absorption	Low	Low	High	High
BBB permeant	No	No	No	No
P-gp substrate	No	No	No	No
CYP1A2 inhibitor	No	No	No	No
CYP2C19 inhibitor	No	No	No	No
CYP2C9 inhibitor	No	No	No	No
CYP2D6 inhibitor	No	No	No	No
**Toxicity**				
Reverse Mutation Assay AMES Test	Non-Toxic	Non-Toxic	Non-Toxic	Non-Toxic
Carcinogens	No	No	No	No
Cytotoxicity	Non-Cytotoxic	Non-Cytotoxic	Non-Cytotoxic	Non-Cytotoxic
Mutagenicity	No	No	No	No

## Data Availability

Data is contained within the article and Supplementary Materials.

## Supplementary Materials

The following supporting information can be downloaded at: https://www.mdpi.com/article/10.3390/ph15040414/s1, Supplementary File, Table S1: Interaction analysis of docked complexes.
