# Supplementary material for: Integrating Network Pharmacology and Molecular Docking Approaches to Decipher the Multi-Target Pharmacological Mechanism of Abrus precatorius L. Acting on Diabetes
Source: Pharmaceuticals (Basel). 2022 Mar 29;15(4):414. doi: 10.3390/ph15040414 (PMC9029140; doi:10.3390/ph15040414)

Table S1: Interaction analysis of docked complexes

Abrisapogenol J	2az5	Abrusin

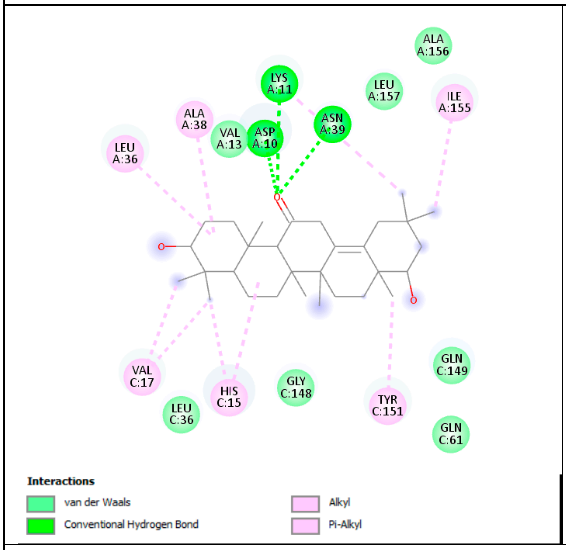

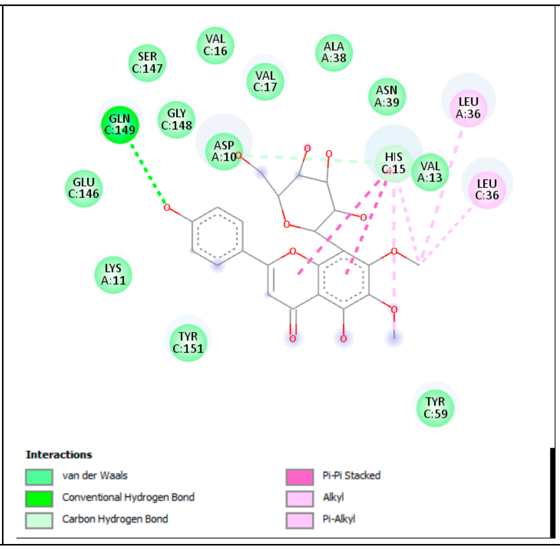

Cycloartenol	2zoq	Precatorine

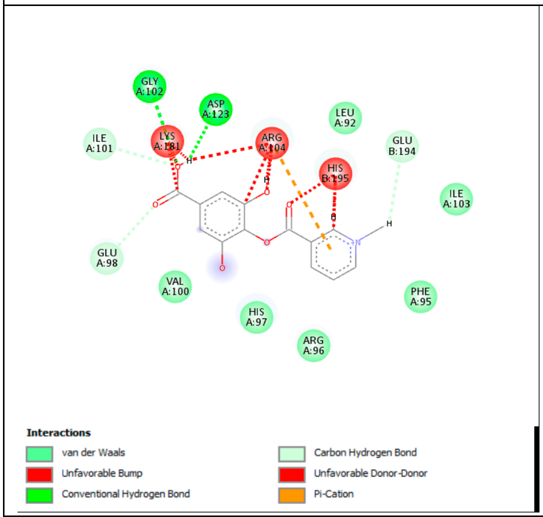

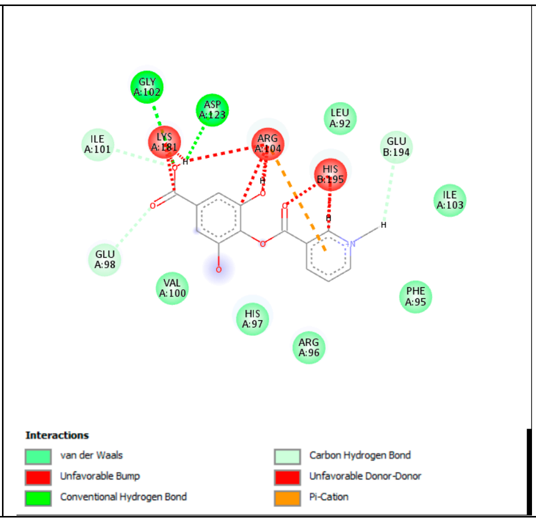

Abrisapogenol J	3qkk	Abrusin

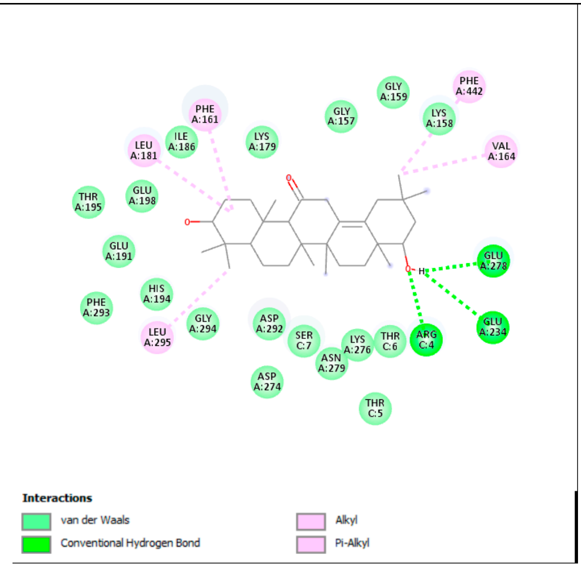

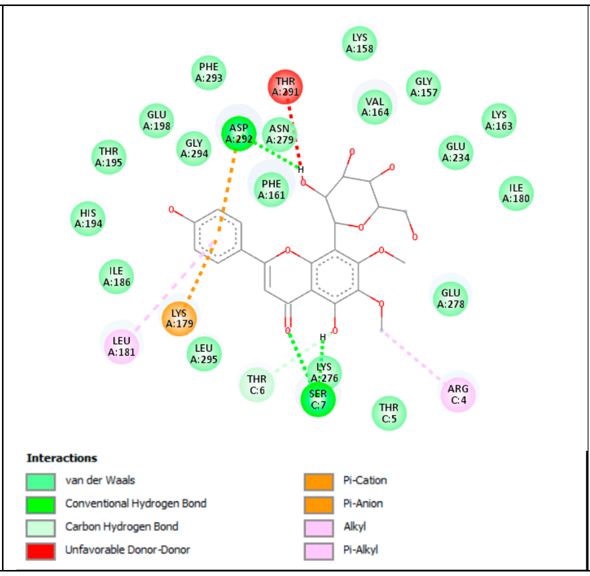

Abrusin | 4iz5 | **Cvcloartenol**

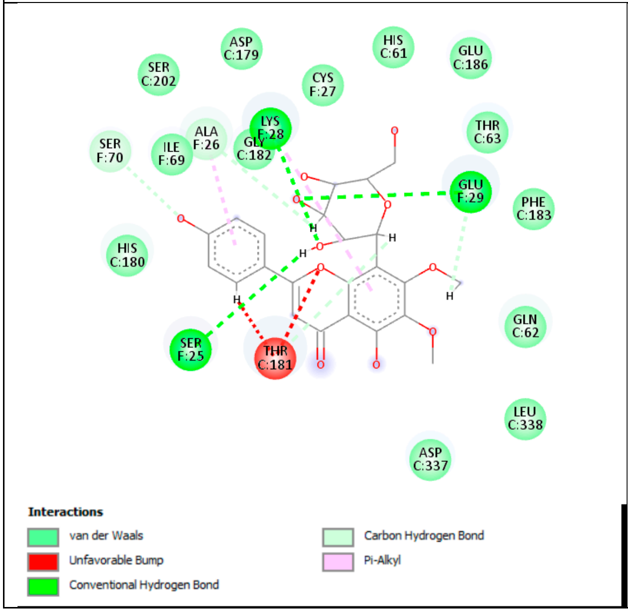

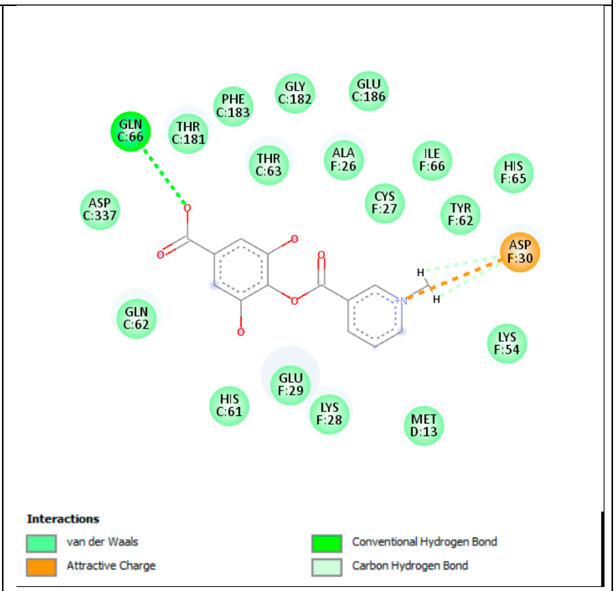

Supplement: Supplementary file 1 [file pharmaceuticals-15-00414-s001.zip › pharmaceuticals-1600645-supplementary.pdf]
